# *RNF2* Missense Variants Disrupt Polycomb Repression and Enable Ectopic Mesenchymal Lineage Conversion During Human Neural Differentiation

**DOI:** 10.21203/rs.3.rs-7143352/v1

**Published:** 2025-08-11

**Authors:** Charles W. Ryan, Samantha L. Regan, Jason B. Sheingold, Anupam Goswami, Maureen Mulhern, Jonathan Ploeger, Samuel Huang, Verity Hartill, Alyssa Rippert, Elizabeth Bhoj, Wendy K. Chung, Jennifer Bain, Kinshuk Raj Srivastava, Stephanie L. Bielas

**Affiliations:** 1 Cellular and Molecular Biology Program, University of Michigan Medical School, Ann Arbor, MI, 48109-5618, USA.; 2 Medical Science Training Program, University of Michigan Medical School, 3703 Med Sci II, 1241 E. Catherine St., Ann Arbor, MI, 48109-5618, USA.; 3 Department of Human Genetics, University of Michigan Medical School, 3703 Med Sci II, 1241 E. Catherine St., Ann Arbor, MI, 48109-5618, USA.; 4 Medicinal and Process Chemistry Division, CSIR-Central Drug Research Institute, Lucknow, U.P., India; 5 Department of Neurology, Columbia University Medical Center, Columbia University, New York, NY, USA; 6 Marshfield Medical Center, Marshfield Clinic Health Center, Marshfield, WI, USA; 7 Department of Clinical Genetics, Chapel Allerton Hospital, Leeds Teaching Hospitals, NS Foundation Trust, Leeds, UK; 8 Leeds Institute of Medical Research, University of Leeds, Leeds, UK; 9 Division of Human Genetics, Children’s Hospital of Philadelphia, Philadelphia, PA, USA; 10 Department of Pediatrics, Boston Children’s Hospital, Harvard Medical School, Boston, MA, USA.; 11 Department of Pediatrics, University of Michigan Medical School, Ann Arbor, MI, 48199-5618, USA.; 12 Neuroscience Graduate Program, University of Michigan Medical School, Ann Arbor, MI, 48109-5618, USA.

## Abstract

Polycomb Repressive Complex 1 (PRC1) catalyzes H2AK119ub1 to facilitate transcriptional repression during development. *De novo* dominant missense variants in *RNF2*, the principal E3 ligase of PRC1, are the genetic basis of Luo-Schoch-Yamamoto syndrome. To investigate the developmental impact of catalytically impaired RNF2 alleles, we engineered hESC lines harboring homozygous hypomorphic *RNF2* missense alleles (*RNF2*^*MS/MS*^) that stably expresses RNF2 but results in reduced H2AK119ub1. Upon directed neural differentiation, *RNF2*^*MS/MS*^ cells exhibited asynchronous neural differentiation and ectopic emergence of mesenchymal fated lineages. Single-cell transcriptomic analyses revealed a fate bifurcation characterized by derepression of *TWIST1* and other epithelial-to-mesenchymal transition (EMT) gene-network components, coinciding with focal loss of H2AK119ub1 and H3K27me3. These findings demonstrate that RNF2-mediated H2AK119ub1 is required to constrain lineage fidelity by repressing context-inappropriate developmental programs during early human neural differentiation and reveal a shared chromatin-based mechanism linking *RNF2* missense variants to both neurodevelopmental pathology and oncogenic plasticity.

## Introduction

Polycomb group (PcG) proteins are essential regulators of transcriptional repression during development, maintaining cell identity and lineage fidelity by silencing lineage-inappropriate gene expression programs [1–4]. Polycomb-mediated repression is achieved through the coordinated activity of histone modifications and higher-order chromatin organization [5–8]. Polycomb Repressive Complex 1 (PRC1) catalyzes monoubiquitination of histone H2A at lysine 119 (H2AK119ub1), a repressive chromatin mark that cooperates with the histone methyltransferase activity of Polycomb Repressive Complex 2 (PRC2), which deposits H3K27me3 [4, 9]. These complexes function in parallel and in concert to reinforce gene silencing at target loci [1, 6–8, 10, 11]. PRC1-mediated H2AK119ub1 is dynamically regulated by the Polycomb Repressive Deubiquitinase complex (PR-DUB), which removes H2AK119ub1 [12]. This dynamic exchange of histone deposition and removal is critical for the precise temporal and spatial regulation of gene expression programs that govern cellular identity and developmental transitions [13].

During early brain development, a diverse array of cell lineages is specified from the neuroepithelium of the rostral neural tube. Multipotent neural progenitor cells (NPCs) residing in the neuroepithelium give rise to the full complement of excitatory neurons that populate the cerebral cortex [14]. In contrast, cranial neural crest (CNC) cells delaminate from the dorsal neural tube through an epithelial-to-mesenchymal transition (EMT), a process initiated by transcription factors such as TWIST1 [15]. TWIST1 initiates signaling cascades that remove epithelial polarity and adherens junctions, enabling CNC cells to migrate and differentiate into mesenchymal derivatives, including bone, cartilage, and connective tissue of the face and skull [16, 17]. Misregulation of this lineage decision-making process has been implicated in neurodevelopmental disorders (NDDs) and contributes to common clinical features such as intellectual disability, epilepsy, and craniofacial malformations [15].

PRC1 complexes are molecularly heterogeneous, consisting of various protein compositions that exhibit dynamic and partially overlapping expression patterns during development [2, 18–20]. All PRC1 complexes share a conserved catalytic core composed of the E3 ubiquitin ligase RNF2 [encoded by *RNF2* (human) and *Ring1b* (mouse)] and its paralog RING1 [encoded by *RING1* (human) and *Ring1a* (mouse)], with RNF2 serving as the primary catalytic subunit during development [18]. The RING and RAWUL domains convey two main RNF2 functions. The C-terminal RAWUL domain of RNF2 mediates interaction with Polycomb group ring finger (PCGF) proteins, such as BMI, required for their incorporation into PRC1, while its N-terminal RING domain facilitates nucleosome binding and catalysis [2, 18]. PRC1 complexes monoubiquitinate H2A and participate in higher-order chromatin organization at developmental gene targets. Both the loss of enzymatic function and disrupted complex assembly compromise Polycomb repression required for cell fate specification [21–26].

Missense variants are a growing category of pathogenic variants being identified through both clinical and research genomic sequencing efforts [27]. Unlike truncating or canonical loss-of-function (LOF) variants, missense variants often affect highly conserved residues and can preserve protein expression. Hypomorphic or hypermorphic proteins encoded by missense alleles, often impact discrete protein function to offer unique mechanistic insights obscured in LOF models displaying broader aberrations. In the context of multi-subunit complexes, they may also function via dominant-negative or gain-of-function mechanisms [28]. Pathogenic *RNF2* missense variants have recently emerged as causative alleles in individuals with neurodevelopmental disorders, including Luo-Schoch-Yamamoto syndrome (LUSYAM; OMIM: 619460) [29]. These variants cluster in evolutionarily conserved regions of *RNF2* that impair protein function, yet their specific impact on human neural development remains unknown. Investigating *RNF2* missense variants can illuminate previously unrecognized protein function, particularly in developmentally regulated and disease-relevant contexts.

In this study, we identify novel *RNF2* variants for LUSYAM. We examine the molecular and neural developmental consequences of a catalytically inactive *RNF2* missense variant (MS). We show that *RNF2*^*MS/MS*^ cells exhibit impaired neural commitment, precocious neuronal differentiation, and the emergence of a distinct mesenchymal cell population during neural differentiation of hESC lines. Single-cell RNA sequencing reveals a bifurcation of cell fate trajectories, with *RNF2*^*MS/MS*^ cells adopting either canonical dorsal forebrain identities or CNC mesenchymal fates marked by EMT-associated transcription factors including *TWIST1*. Chromatin profiling demonstrates concordant loss of H2AK119ub1 and H3K27me3 at Polycomb target loci, leading to derepression of *TWIST1* and its downstream effectors. Together, these findings reveal a critical function for RNF2-dependent H2AK119ub1 in suppressing EMT during neural differentiation and maintaining lineage fidelity. Our study provides mechanistic insight into how *RNF2* missense variants contribute to neurodevelopmental disorders and implicates Polycomb dysregulation as a shared epigenetic mechanism in both developmental pathology and metastasis of carcinoma cells.

## Results

### *RNF2* missense variants are associated with neurodevelopmental disorders and cancer

**Figure 1:**
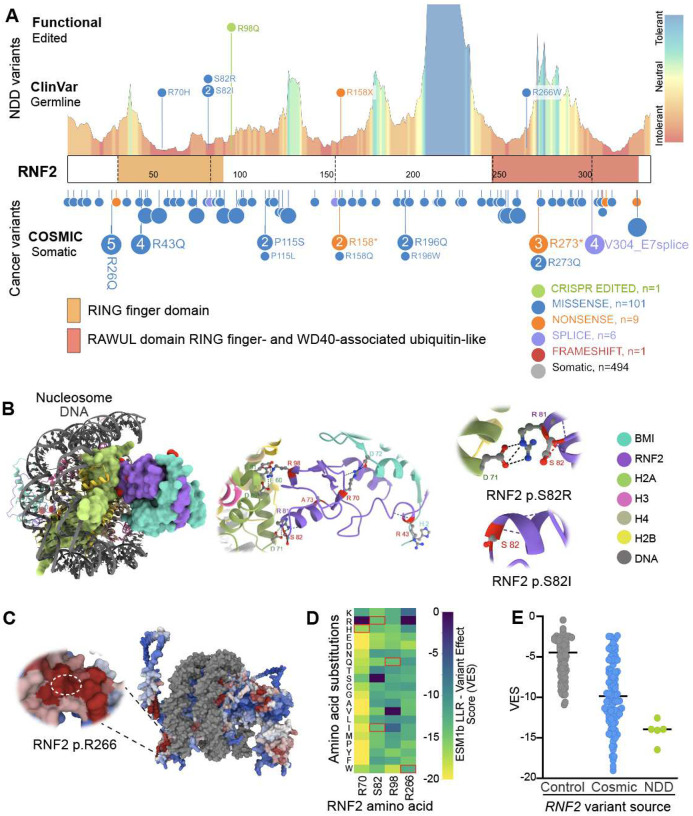
Pathogenic RNF2 missense variants are associated with neurodevelopmental phenotypes with overlapping features. **(A)** MetaDome plot of RNF2 depicting pathogenic missense variants reported in Table 1 are clustered around high constraint regions of RNF2. RNF2 variants (bottom) deposited in COSMIC cancer database are found distributed across the entirety of the protein. **(B)** cryo-EM structure (PDB: 8PP7) of RNF2 bound to the nucleosome highlighting the contribution of *RNF2* p.R70 in BMI1 binding and the role of *RNF2* S82 in histone 2A binding, adapted from Luo et. al 2021. **(C)** Model of RNF2 bound to the nucleosome demonstrating the participation of RNF2 R98 in an acid:base interaction with the histone 2A tail, adapted from Pierce et. al 2018. **(D)** The variant effect predictions for RNF2 substitutions. The variant effect score (VES) is the ESMb1 log likelihood ratio (LRR) of effect scores. **(E)** COSMIC RNF2 variant log likelihood ratio scores (Control average = −4.91, COSMIC average = −9.732, Neurodevelopmental disorders (NDD) average= −14.192).

*RNF2* missense variants are enriched in both NDDs and cancer, underscoring their pathogenic potential [29, 30]. Clinical and research-based human genetic evaluations identified multiple *de novo* variants that impinge on evolutionarily conserved and highly constrained RNF2 amino acid residues (Fig. 1A, **S1A**). In compliance with institutional review board approved recruiting protocols, we identified 3 monoallelic *RNF2* variants in 4 unrelated individuals: c.245G>T (p.S82I) (identified in two unrelated individuals), c.796A>T (p.R266W), and c.472C>T (p.R158*) (Table 1, Fig. 1A). These variants resemble the *RNF2* c.209G>A(p.R70H) and c.246T>G(p.S82R) variants that were originally identified as the genetic basis of LUSYAM [29]. Recurrent *de novo* variants and/or those affecting the same RNF2 amino acids implicate a critical functional role. An example of this is the variants disrupting RNF2 pS82 (p.S82I and p.S82R), which reveal a particular sensitivity of this amino acid to substitutions. Population level genetic data indicates that *RNF2* is highly constrained. *RNF2* missense variants are underrepresented in control human genome data (gnomAD Z = 2.97). Constraint analyses, including MetaDome, also indicate that amino acid substitutions are not tolerated, particularly in functional domains (Fig. 1A). Additionally, *RNF2* missense variants are highly enriched in the COSMIC cancer database, and, like NDDs, it predominantly features missense rather than truncating mutations, highlighting pathogenic mechanisms of *RNF2* missense variants yet to be revealed.

The *RNF2* c.472C>T (p.R158*) variant was the first monoallelic nonsense variant associated with LUSYAM (inheritance data are unavailable). This variant is notable, as *RNF2* is highly intolerant to LOF variants with a LOF observed / expected upper bound fraction (LOEUF) score of 0.15 as calculated by gnomAD specifications. This metric supports the pathogenicity of the p.R158* nonsense variant and the dosage-sensitivity of *RNF2*. The *RNF2* c.472C>T (p.R158*) variant is on the N-terminal border of exon 4 within the main transcript (ENST00000367510.8) but is not predicted to disrupt splicing. Exon 4 is the final exon of a minor RNF2 isoform (ENST00000367509.8). In this isoform, mRNA expressing the *RNF2* c.472C>T (p.R158*) variant would escape nonsense mediated decay, which could produce a truncated protein that partially rescues the haploinsufficiency consistent with a LOF genetic mechanism. This same variant is present in sequencing data from both NDD cohorts and COSMIC samples but is not observed in unaffected controls (gnomAD v3.1.2).

*RNF2* variants are associated with overlapping neurodevelopmental phenotypes (Table 1). All individuals in the *RNF2* cohort presented with prenatal complications (6/6). Half had intrauterine growth restriction (3/6) and one was small for gestational age (1/6). Oligohydramnios (1/6) and polyhydramnios (1/6) were noted. All individuals had gastrointestinal and feeding difficulties (6/6). Gastroesophageal reflux (3/6), dysphagia (2/6), constipation (1/6), and dysmotility (1/6) associated with failure to thrive (4/6) that required feeding tube support (4/6). All individuals presented with craniofacial dysmorphisms, primarily affecting the mouth (4/6), eyes (5/6), and ears (3/6). Shared features include strabismus (4/6), almond-shaped eyes (3/6) and hypertelorism (2/6). Synophrys was present in 2 individuals (2/6). Recurrent and/or chronic otitis media (4/6) were experienced by the majority of individuals, while hearing loss was less frequent (2/6). Cardiovascular (4/6), renal (2/6), and respiratory (2/6) anomalies were also described.

Neurological symptoms were present in all individuals (6/6), including hypotonia (5/6), seizures (3/6), and spasticity (2/6). Delayed myelination on brain MRI was noted (2/6), with myelination improved, but not fully normal in one individual by 8 months of age and improved to within normal limits for a different individual by 12 months of age. Many individuals experienced developmental delay and intellectual disability (5/6). A minority of individuals also displayed microcephaly (2/6), plagiocephaly (2/6), and a flat occiput (1/6). Individual 6, who carries a truncating *RNF2* variant, was diagnosed with a learning disability. Individual 6 is the only individual to present with hemifacial microsomia, 5^th^ finger clinodactly, preauricular tags, complex tethered cord with lipomyelomeningocele, Chiari I malformation, hypoplastic left cochlea with congenital unilateral hearing loss, horizontal gaze palsy, congenital scoliosis with hemi-vertebrae at T6 and butterfly vertebrae at L3, torticollis, horseshoe kidney, and high pain tolerance.

### RNF2 substitutions reflect critical PRC1 functions

RNF2 is critical for PRC1 complex integrity, nucleosome binding and H2A ubiquitination [2, 29, 31]. Modeling based on the previously published cryo-EM structure of a PRC1 bound nucleosome allowed predicted-pathogenic RNF2 amino acid substitutions to be interrogated (**Fig. S1C**) [2]. This analysis indicated that RNF2 p.R70H, p.S82R, p.S82I and p.R266W amino acid substitutions disrupt PRC1 functions through distinct biochemical mechanisms (Fig. 1B). RNF2 p.R70H alters the affinity of RNF2 for the acid patch of H2A, which is required for lysine 119 ubiquitination. RNF2 p.S82I and p.S82R substitutions disrupt the same amino acid but with different molecular effects. Similar to RNF2 p.R70H, the RNF2 p.S82R variant disrupts interaction between RNF2 and the H2A acid patch, while the RNF2 p.S82I substitution is predicted to disrupt the bond with its neighbouring residue, consequently affecting RNF2 stability (Fig. 1B). The RNF2 ubiquitin-like RAWUL domain is required for dimerization with BMI1 [2]. According to MetaDome constraint data, the RNF2 R266W substitution is constrained and required for this function (Fig 1A). ScanNet was used to identify the amino acids in the RAWUL domain with high probability to mediate protein-protein interactions [32]. Based on this analysis the RNF2 R266W variant is predicted to impinge on RAWUL domain protein interactions (Fig. 1C).

*In silico* analysis using computational variant effect prediction tools were used to compare NDD *RNF2* missense variants. ESMb1 workflow is a large language model that predicts pathogenicity based on amino acid sequence, rather than population genetic resources [33]. Nevertheless, ESM variant effect scores (VES) and MetaDome pathogenicity predictions converge on a similar subset of RNF2 amino acids that overlap with RNF2 functional domains (**Fig. S1B**). The NDD *RNF2* missense variants all generate a negative ESMb1 log-likelihood ratio VES of −14.28 out of a range of 0.00 to −20.00, consistent with a pathogenic effect (Fig. 1D, Fig. 1E). Substitution of RNF2 S82 for arginine and isoleucine resulted in scores of −14.06 and −13.95, respectively, emphasizing the pathogenic outcomes that can be caused by missense variants.

The pathogenicity of *RNF2* missense variants is also supported by the enrichment of *RNF2* missense variants in the COSMIC cancer database, as opposed to termination variants [34]. NDD *RNF2* missense variants were also compared to COSMIC variants and top 50 control missense variants with the highest allele frequency in gnomAD (**STable 1**). RNF2 NDD substitutions exhibit as range of VESs from −12.64 to −16.46 with an average of −14.28. COSMIC missense variant (92/112) VESs range from −2.391 to −19.02 with an average of −9.73 (Fig. 1E). Consistent with the intolerance of RNF2 to LOF, NDD or cancer substitutions did not exhibit the most pathogenic VES of −20.00, highlighting the contribution of hypomorphic *RNF2* missense variants to the pathology of NDDs and cancer (Fig. 1E).

### Hypomorphic RNF2^MS^ diverts *in vitro* neural differentiation

**Figure 2:**
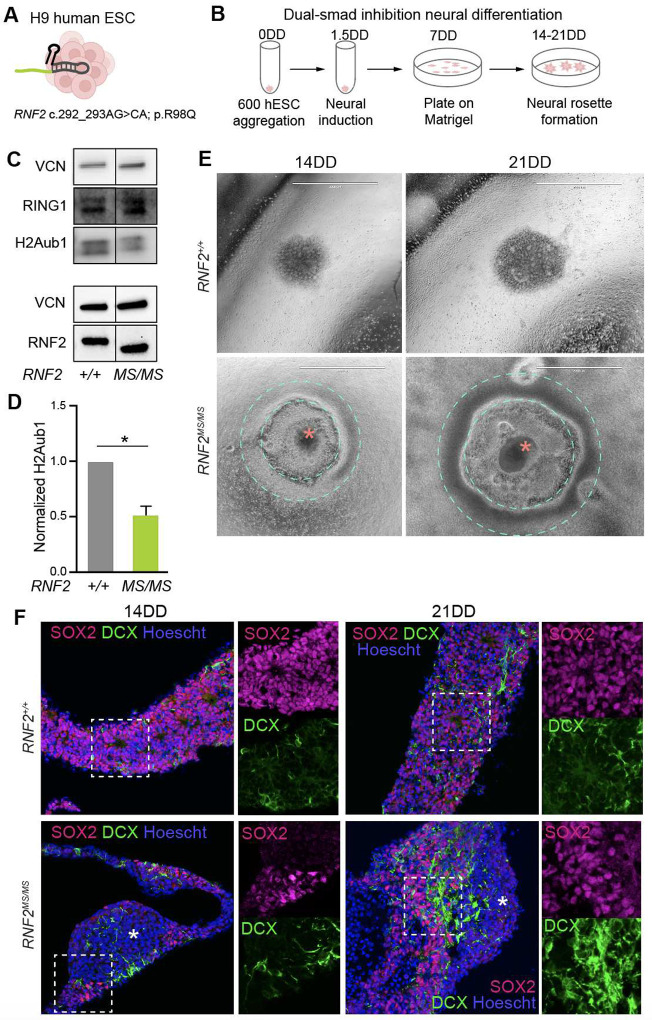
*RNF2*^*MS/MS*^ hESCs exhibit abnormal neural differentiation. **(A)** *RNF2* c.292_293AG > CA;p.R98Q was introduced into isogenic hESCs via CRISPR-Cas9 genome editing. **(B)** hESCs were subjected to 14 or 21 days of dual-SMAD inhibition protocol to generate neural rosettes. **(C)** Western blot depicting RING1, RNF2, and H2AK119ub1, with Vinculin as a loading control, n= 4 biological replicates. This image was adapted from Ryan et al., 2024. **(D)** Quantification of H2AK119ub1 levels normalized to Vinculin. Data represent the mean signal relative to wild type ± SEM. *95% CI does not intersect fold change of 1 relative to wild type, n= 4 biological replicate western blots per genotype. This analysis was adapted from Ryan et al., 2024. **(E)** Visualization of *RNF2*^+/+^ and *RNF2*^*MS/MS*^ neural rosettes at day 14 and day 21 of differentiation using an EVOS light microscope. **(F)** Representative immunohistochemistry showing SOX2 (magenta), DCX (green), and Hoescht (blue) immunostaining of *RNF2*^+/+^ and *RNF2*^*MS/MS*^ neural rosettes at day 14 and 21 of differentiation. Tissue where SOX2 and DCX is absent is denoted with asterisks. Insets are highlighting differences in SOX2 and DCX expressing cells in *RNF2*^+/+^ and *RNF2*^*MS/MS*^ neural fated tissues. This experiment was repeated 3 times with similar results.

To investigate the molecular pathology of *RNF2* missense (*RNF2*^*MS*^) alleles, we generated homozygous *RNF2* c.292_293AG > CA;p.R98Q hESC lines (*RNF2*^*MS/MS*^) using CRISPR/Cas9 genome editing (Fig. 2A) [2, 31, 35]. Based on structural and functional studies, RNF2 R98 is necessary for efficient H2AK119ub1 catalysis, but is not predicted to impact PRC1 complex formation. The basic side chain of R98 inserts into the H2A acid patch, allowing for the electrostatic interactions required for H2A ubiquitination, a mechanism implicated for pathogenic LUSYAM *RNF2* missense variants (Fig. 1B) [2, 29]. The RNF2 R98Q substitution is highly intolerant to change according to control population data, and has an ESM1b VES score of −14.00, in line with LUSYAM variants. To determine the pathogenic effect of the *RNF2*^*MS*^ allele on neural development, *RNF2*^*MS/MS*^ and isogenic control hESC lines were neural differentiated to a dorsal forebrain fate using a dual SMAD inhibition protocol to generate neural rosettes and neural progenitor cells (NPCs) (Fig. 2B). Stable expression of RNF2^MS^ was detected in NPCs by western blot at a level comparable to RNF2 in control cells (Fig. 2C) [31]. Nevertheless, *RNF2*^*MS/MS*^ NPCs exhibit an ~50% reduction in whole-genome H2AK119ub1 levels by western blot (Fig. 2C–D) [31]. CUT&RUN analysis of RNF2 revealed that RNF2^MS^ genome-wide occupancy overlaps with that of RNF2 and its paralog RING1, suggesting that catalytically compromised RNF2^MS^ competes for PRC1 target sites, hindering PRC1 H2AK119ub1 ligase function [31].

H2AK119ub1 is required for Polycomb transcriptional repression critical to development, and reduced H2AK119ub1 is a direct outcome of RNF^MS^ expression. To investigate the impact of reduced H2AK119ub1 on development, 3D tissue from neural differentiation of *RNF2*^*MS/MS*^ and isogenic control hESC lines was analyzed. During dual SMAD neural differentiation, embryoid bodies plated on Matrigel form neural rosettes between 10–14 days of differentiation (DD), which we observed with control hESC lines. However, a unique, fully penetrant cytoarchitectural phenotype was observed by light microscopy in the *RNF2*^*MS/MS*^ cultures (Fig. 2E). During dual SMAD neural differentiation, *RNF2*^*MS/MS*^ embryoid bodies formed abnormally large rosette mounds, when cultured on Matrigel (Fig. 2E). In addition, of a raised perimeter of cells formed that encompassed the mound of neural rosettes by 14DD, which increases in prominence through 21DD (**Fig. S2A,B**). To assess if the *RNF2*^*MS/MS*^ structures generated during neural differentiation were composed of neural fated cells, the tissue was fixed, cryosectioned, and stained for the NPC marker SOX2 and immature neural marker DCX by immunohistochemistry (Fig. 2F). Consistent with their abnormal appearance, a distinct proportion of the *RNF2*^*MS/MS*^ tissue did not express SOX2 or DCX, while *RNF2*^+/+^ tissue was almost entirely composed of SOX2 or DCX positive cells (Fig. 2F). In the region where SOX2 and DCX was present in *RNF2*^*MS/MS*^, there was an increase in DCX expression, consistent with a change in the timing of differentiation. This suggests RNF2-dependent H2AK119ub1 is necessary for high fidelity and timing of differentiation from hESCs to NPCs and neurons.

### Mesenchymal fated cells emerge during *RNF2*^*MS/MS*^ neural differentiation

**Figure 3:**
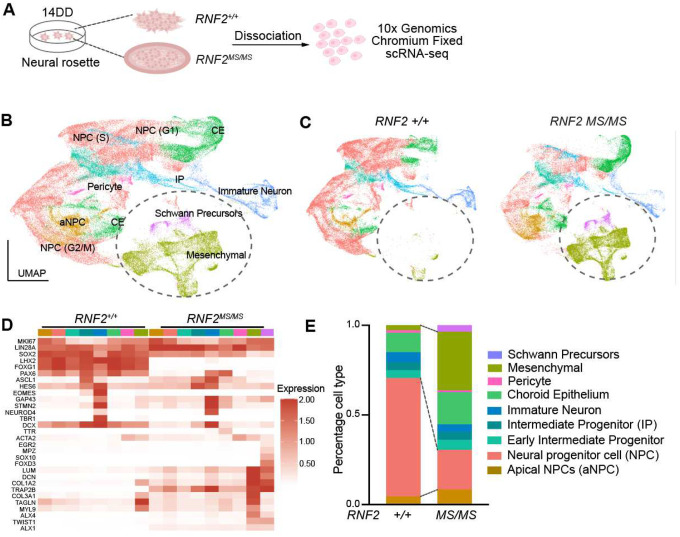
*RNF2*^*MS/MS*^ hESCs give rise to ectopic mesenchymal fates during early neurogenesis **(A)** *RNF2*^+/+^ and *RNF2*^*MS/MS*^ neural rosettes at day 14 of differentiation were dissociated and subjected to single cell RNA sequencing. **(B)** Projection of scRNAseq data onto UMAP space with clusters annotated by expression of canonical markers. **(C)** UMAP plots with *RNF2*^+/+^ and *RNF2*^*MS/MS*^ data projected separately. **(D)** Gene expression heatmap demonstrating expression of canonical markers across cell types by genotype. Columns represent cell types (color coded according to colors in UMAP plot) split by genotype (wild type = +, *RNF2*^*MS/MS*^ = MS). **(E)** Bar chart demonstrating the proportional representation of each cell type within *RNF2*^+/+^ and *RNF2*^*MS/MS*^ tissue, data are represented as mean ± SEM (*n* = 3 *RING1*^+/+^ and *n* = 4 *RING1*^*MS/MS*^ independently differentiated biological replicates).

To characterize the cellular composition of the structures generated during *RNF2*^*MS/MS*^ neural differentiation, we performed single cell RNA (scRNA) sequencing of tissue at 14DD of the dual-SMAD differentiation protocol, soon after the highly penetrant neural rosette-encompassing ring of cells starts to accumulate in *RNF2*^*MS/MS*^ cultures (Fig. 3A). The data from three replicates of each *RNF2* genotypes were integrated, projected onto low dimensional space, and clustered using Seurat (v5.0.1) (**Fig. S2A, Fig. S2B**) [36]. This revealed the expected neural fated cell types, including proliferative neural progenitor cells, intermediate progenitors, and immature neurons (Fig. 3B). The appearance of mesenchymal lineage cells and Schwann cell precursors, however, was unexpected based on the neural differentiation protocol used. To assess the genotype-dependent component of this phenotype, the clusters were projected according to genotype. The mesenchymal linages were exclusively present in *RNF2*^*MS/MS*^ tissue (Fig. 3C). Each cluster was annotated based on multiple mRNAs that define distinct cell types. A heatmap of the average expression of canonical cell type markers was plotted to visualize their expression in control and *RNF2*^*MS/MS*^ tissue, revealing markers of dorsal forebrain neural lineage in both genotypes and mesenchymal markers in *RNF2*^*MS/MS*^ samples only (Fig. 3D). Interestingly, expression of mesenchymal cell type markers was also observed at low levels in the neural cell types within the *RNF2*^*MS/MS*^ tissue (Fig. 3D). This suggests a failure to suppress mesenchymal gene expression that ultimately enabled a subset of tissue to convert to an alternate cell fate. Transformed cell types make up a significant fraction of *RNF2*^*MS/MS*^ tissue, with mesenchymal and Schwan cell precursor cells representing 33% and 4% of the tissue, respectively, compared to just 3% and 0% in control tissue (Fig. 3E). NPCs comprise a far smaller fraction of 22% in *RNF2*^*MS/MS*^ tissue, compared to 66% of *RNF2* controls (Fig. 3E).

### *RNF2*^*MS/MS*^ mesenchymal cells arise through a developmental trajectory distinct from neural lineages

**Figure 4:**
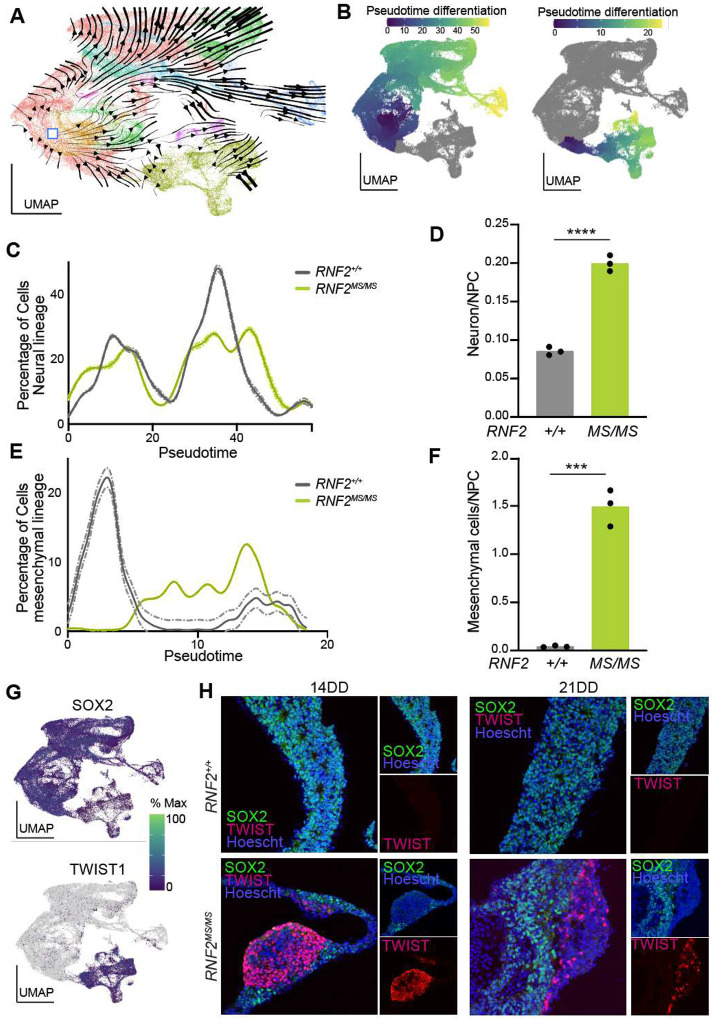
Mesenchymal fates in differentiating *RNF2*^*MS/MS*^ hESCs follow expression of lineage master regulators. **(A)** UMAP plot of RNA velocity, depicting a bifurcation of lineages from ESCs into mesenchymal and neural cell lineages. **(B)** UMAP plots with pseudotime trajectory, depicting two separate paths of the neural and mesenchymal cell lineages. **(C)** Density plot demonstrating the proportion of cells in *RNF2*^+/+^ and *RNF2*^*MS/MS*^ at each point along the neural pseudotime trajectory. The mean ± SEM (dashed line) are drawn for *RNF2*^+/+^ and *RNF2*^*MS/MS*^ neural rosettes. **(D)** Quantification of the neuron to NPC ratio in *RNF2*^+/+^ and *RNF2*^*MS/MS*^ tissue. Each data point represents the ratio of a single replicate, n=3. ****Student’s T-test *p* < 0.0001. **(E)** Density plot demonstrating the proportion of cells in *RNF2*^+/+^ and *RNF2*^*MS/MS*^ at each point along the mesenchyme pseudotime trajectory. The mean ± SEM (dashed line) are drawn for *RNF2*^+/+^ and *RNF2*^*MS/MS*^ neural rosettes. **(F)** Quantification of the mesenchymal cell to NPC ratio in *RNF2*^+/+^ and *RNF2*^*MS/MS*^ tissue. Each data point represents the ratio of a single replicate, n=3. ***Student’s T-test *p* < 0.001. **(G)** UMAP plots with cells colored by their average SOX2 (left) and TWIST1 (right) expression level. **(H)** Representative immunohistochemistry showing SOX2 (green), TWIST1 (red), and Hoescht (blue) immunostaining of *RNF2*^+/+^ and *RNF2*^*MS/MS*^ neural rosettes at days 14 and 21 of differentiation. This experiment was repeated 3 times with similar results.

The generation of mesenchymal cells during *RNF2*^*MS/MS*^ neural differentiation suggests that decreased H2AK119ub1 permits expression of alternative fate gene expression programs. To identify the transcriptomic determinants of mesenchymal fate, RNA velocity was used to create a time-dependent relationship of transcriptomes by comparing the balance of spliced versus unspliced mRNA abundance between clusters to infer paths of differentiation [37]. This analysis revealed two major paths of differentiation starting from a population of multipotent progenitor cells (Fig. 4A). Neural differentiation was traced through NPCs and intermediate progenitors to neurons, consistent with the dorsal forebrain differentiation trajectory established by dual SMAD inhibition. A second trajectory progressed from multipotent progenitors along mesenchymal transcriptional states characterized expression by of TWIST1 [15].

The synchrony of the neural and mesenchymal differentiation trajectories by genotype were evaluated by performing pseudotime differentiation of clusters along these distinct trajectories (Fig. 4B, **Fig. S3A**) [38, 39]. *RNF2*^*MS/MS*^ neural differentiation exhibits a forward shift, consistent with an asynchronous differentiation biased towards a more mature fate (Fig. 4C). A similar effect on the timing of neural differentiation is implicated by the earlier expression of GAP43 relative to SOX2 along the pseudotime trajectory of *RNF2*^*MS/MS*^ neural differentiation compared to the control (**Fig. S3B**). While neurons represented a small percentage of both samples at this early stage of differentiation (4.4% vs 5.7%), the neuron to NPC ratio was significantly higher in *RNF2*^*MS/MS*^ tissue, reflective of such asynchronous differentiation (*p* < 0.0001) (Fig. 4D).

Along the neural crest mesenchymal trajectory, transcriptional dynamics revealed that reduced expression of multipotency marker SOX2 preceded differential expression of transcription factors involved in establishing mesenchymal fates (TWIST1, ALX1, ALX4, ETS1, PAX3, TFAP2B and MSX2), with TWIST1 being foremost among them, and several of their targets (ABCA9, PCOLCE, DDR2, and LUM) in the transcriptional network (**Fig. S3C**). Control multipotent progenitors did not progress along the mesenchymal trajectory, as depicted by pseudotime differentiation analysis (Fig. 4E). The tendency of *RNF2*^*MS/MS*^ cells to differentiate along the mesenchymal trajectory under directed neural differentiation conditions is demonstrated by the significantly higher mesenchymal cell to NPC ratio observed in *RNF2*^*MS/MS*^ tissue (*p* = 0.0002) (Fig. 4F).

There is an inverse relationship between the expression of *SOX2* and the mesenchymal master regulator *TWIST1* (Fig. 4G). Indeed, *TWIST1* is almost exclusively expressed in the mesenchymal cell clusters, where *SOX2* expression is lowest (Fig. 4G). Immunostaining of *RNF2*^+/+^ and *RNF2*^*MS/MS*^ tissue at 14 and 21 DD with SOX2 and TWIST1 validated this distinct expression profile at the protein level. A subset of *RNF2*^*MS/MS*^ cells robustly express TWIST1 at the expense of SOX2 at 14DD and 21DD, but no TWIST1 cells were detected in *RNF2*^+/+^ tissue (Fig. 4H). Cytoarchitecturally, TWIST1 expressing cells are peripherally enriched around centrally located SOX2 expressing neural rosettes in *RNF2*^*MS/MS*^, implicating distinct cellular and developmental dynamics of TWIST1 positive cells (**Fig. S2C**). These findings demonstrate that RNF2 dependent H2AK119ub1 is necessary to suppress ectopic expression of mesenchymal fate-specific gene expression programs.

### Reduced H2AK119ub1 enables ectopic expression of pioneer transcription factors and fate profiles

To investigate the contribution of reduced H2AK119ub1 on the ectopic expression and transformation of mesenchymal cells in *RNF2*^*MS/MS*^ tissue, CUT&RUN was performed to profile H2AK119ub1 and H3K27me3 occupancy at functional domains genome wide. This analysis was performed on 2D cultures of *RNF2*^+/+^ and *RNF2*^*MS/MS*^ NPCs dissociated from neural rosettes at DD14. Analysis across Polycomb target genes revealed a strong correlation between H2AK119ub1 and H3K27me3 (**Fig. S4A**). This correlation between H2AK119ub1 and H3K27me3 at PRC1 target genes persisted in both control and *RNF2*^*MS/MS*^ tissue (**Fig. S4B)**. These results demonstrate that H2AK119ub1 and H3K27me3 act in concert in NPCs.

**Figure 5:**
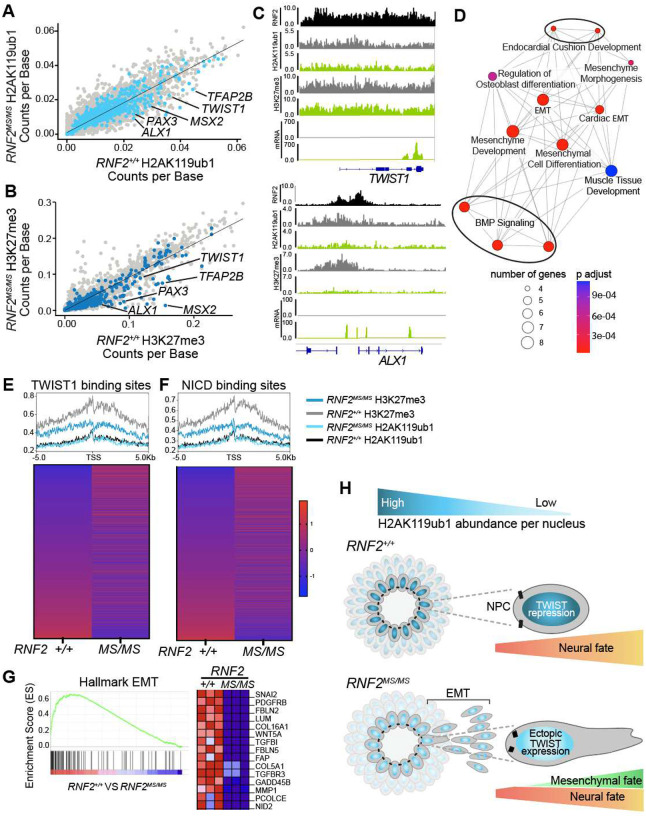
Reduced H2AK119ub1 permits ectopic expression of pioneer transcription factors and fate profiles. **(A)** Correlation between *RNF2*^+/+^ and *RNF2*^*MS/MS*^ H2AK119ub1 occupancy in dissociated *RNF2*^+/+^ neural rosettes, with genes with significantly higher expression in bulk RNA sequencing colored purple, and a subset of genes with the 5% most reduced (5% most negative residual values) H2AK119ub1 occupancy in *RNF2*^*MS/MS*^ labeled. **(B)** Correlation between *RNF2*^+/+^ and *RNF2*^*MS/MS*^ H3K27me3 occupancy in dissociated *RNF2*^+/+^ neural rosettes, with genes with significantly higher expression in bulk RNA sequencing colored purple, and a subset of genes with the 5% most reduced (5% most negative residual values) H3K27me3 occupancy in *RNF2*^*MS/MS*^ labeled. **(C)** RNF2, H2AK119ub1, and H3K27me3 CUT&RUN tracks with bulk RNA sequencing tracks at the *ALX1 and TWIST1* locus. CUT&RUN data are normalized to counts per 10 million mapped reads. Genic signal from two merged biological CUT&RUN replicates. **(D)** Gene ontology pathways that are significantly enriched among the 46 overlapping genes with increased expression, decreased H2AK119ub1, and decreased H3K27me3 in *RNF2*^*MS/MS*^ from Fig. S4A,B. **(E)** Heatmap of average expression of 7670 targets of TWIST1 with a CUT&RUN profile plot showing decreases in both H2AK119ub1 and H3K27me3 around their transcription start sites in *RNF2*^*MS/MS*^ NPCs compared to control. **(F)** Heatmap of average expression of 7670 targets of NOTCH1 with a CUT&RUN profile plot showing decreases in both H2AK119ub1 and H3K27me3 near their transcription start sites in *RNF2*^*MS/MS*^ NPCs compared to controls. **(G)** GSEA enrichment plot and expression heatmap for 15 leading edge genes for the reactome “Hallmark epithelial mesenchymal transition” pathway. Comparison of means between *RNF2*^+/+^ and *RNF2*^*MS/MS*^ are made with unpaired two-tailed t-tests with correction for multiple testing by two-stage step-up method of Benjamini, Krieger, and Yekutieli, Nominal p value = 0.0 and a normalized enrichment score of 2.44. Source data are provided as a Source Data file. **(H)** Representative model demonstrating the loss of H2AK119ub1 permitting ectopic expression of pioneer factors, such as TWIST1, to cause a shift in fate.

While the decrease in H2AK119ub1 in *RNF2*^*MS/MS*^ tissue is observed globally, we hypothesized that genes involved in mesenchymal development would be disproportionately affected. To identify genes with the largest decrease, H2AK119ub1 occupancy was correlated between control and *RNF2*^*MS/MS*^ tissue across all genes. The number of H2AK119ub1 read counts were normalized by gene length, and a linear model was used to generate an expected count in *RNF2*^*MS/MS*^ tissue based on the count in *RNF2*^+/+^ tissue. For any given gene, subtracting the linear model’s expected value from the experimentally observed count in *RNF2*^*MS/MS*^ produces the gene’s ‘residual value’— the deviation in the *RNF2*^*MS/MS*^ H2AK119ub1 level from control at that locus. The five percent of genes with the largest magnitude decreases were noted, including several genes involved in mesenchymal development such as *TWIST1, TFAP2B, ALX1, PAX3, ETS1, MSX2,* and *PCOLCE* (Fig. 5A). Given the observed strong correlation between H2AK119ub1 and H3K27me3, we hypothesized that the genes with the greatest reduction in H2AK119ub1 would also be among those with the greatest reduction in H3K27me3. Calculating H3K27me3 occupancy between *RNF2*^+/+^ and *RNF2*^*MS/MS*^ tissue revealed that all of the aforementioned mesenchymal genes were also in the five percent of genes with the largest H3K27me3 decreases (Fig. 5B).

To relate changes in H2AK119ub1 and H3K27me3 to altered gene expression, bulk RNA sequencing was performed on *RNF2*^+/+^ and *RNF2*^*MS/MS*^ tissue. Consistent with findings from single cell RNA sequencing and as predicted by the decrease in both H2AK119ub1 and H3K27me3, significant upregulation of the same network of genes that determine the mesenchymal lineages in CNC fate was identified (**Fig. S5A**). CUT&RUN and bulk RNA sequencing tracks were used to visualize increased expression coinciding with decreased H2AK119ub1 and H3K27me3 at individual loci of the mesenchymal lineage gene network components (Fig. 5C). RNA sequencing tracks depict the ectopic derepression of these neural crest pioneer transcription factors in *RNF2*^*MS/MS*^ tissue that are not expressed in dorsal forebrain fated NPCs. To identify the upregulated DEGs that had a corresponding disproportionate decrease in local H2AK119ub1 and H3K27me3, the DEGs were intersected with the genes that were in the five percent most decreased H2AK119ub1 and five percent most decreased H3K27me3. Among the 46 overlapping genes, several were members of the CNC mesenchymal lineage gene network, including *TWIST1, TFAP2B, ALX1, PAX3, ETS1 and MSX2* (**Fig. S5B**). Gene ontology (GO) analysis performed on the resulting list revealed enrichment of genes involved in “mesenchyme development” and “mesenchymal cell differentiation” (Fig. 5D). These results reveal the critical functions of PRC1-dependent H2AK119ub1 in repressing expression of mesenchymal fate determinants during human neural differentiation.

To determine how reduced H2AK119ub1 affects transcriptional regulation by key developmental transcription factors, we assessed the Polycomb histone modification landscape at the transcriptional start sites (TSS) of differentially expressed genes with TWIST1 or NOTCH1 intracellular domain (NICD) binding sites. We observed decreased H2AK119ub1 and H3K27me3 occupancy at these TSS in *RNF2*^*MS/MS*^ NPCs, consistent with the global reduction of Polycomb repressive marks (Fig. 5E, F). However, unlike the *TWIST1* locus itself, these loci are not disproportionately affected. Despite this, both TWIST1 and NICD target genes exhibit widespread differential expression, suggesting that the reduction of the Polycomb repressive landscape in *RNF2*^*MS/MS*^ NPCs establishes a permissive chromatin environment that enables activation of CNC-mesenchymal gene networks and contributes to asynchronous neural differentiation (Fig. 5E, F). To determine TWIST1 target gene expression programs altered in *RNF2*^*MS/MS*^ NPCs, we performed gene set enrichment analysis on the complete list of TWIST1 target genes. The most enriched pathway was Hallmark epithelial mesenchymal transition, NES 2.44, FDR p = 0.000 (Fig.5G).

Taken together, these results support a model where an RNF2-dependent reduction in H2AK119ub1 during dorsal forebrain neural differentiation of hESCs allows for ectopic derepression of TWIST1 (Fig.5H). Enhanced by the diminished H2AK119ub1 and H3K27me3 globally, TWIST1 expression activates the transcriptional cascade that propagates the CNC fate and EMT cell biology. Cells that lose attachment to the neural rosette neural epithelium migrate away and accumulate around the aggregates of neural tissue.

## Discussion

This study expands the *RNF2* allelic and phenotypic spectrum of LUSYAM syndrome. We show that RNF2-mediated H2AK119ub1 is essential for maintaining the identity of neural-fated lineages by actively repressing alternative lineage transcriptional programs. Using an *in vitro* model of dorsal forebrain neural differentiation from hESCs, we demonstrate that catalytic impairment of RNF2 disrupts PRC1 function, reduces global H2AK119ub1 levels, and enabled inappropriate acquisition of mesenchymal fates during dorsal forebrain differentiation. These findings demonstrate that loss of RNF2 catalytic activity destabilizes lineage fidelity through derepression of EMT master regulators such as TWIST1, implicating a shared chromatin-mediated mechanism across NDDs and cancer. Together, our genetic and functional data provide critical insight into the role of RNF2 in human development and establish H2AK119ub1 as a key repressive barrier against fate infidelity.

The spectrum of *RNF2*-associated clinical features suggests that *RNF2* missense and nonsense variants may act through distinct pathogenic mechanisms, giving rise to phenotypically divergent RNF2-related NDDs. The shared features between individuals with *RNF2* missense variants, particularly among unrelated individuals with substitutions in the same amino acid, reveal the phenotypic scope and range of severity for similar variants. The missense variant-associated phenotypes that characterized LUSYAM coalesce on ID, seizures, and growth restriction. In contrast, individual 6, who carries the *RNF2* c.472C>T (p.R158*) nonsense variant, overlaps less with the *RNF2* missense-associated phenotype, presenting with a broader and clinically distinct constellation of features, including learning disability, kidney involvement, hemifacial microsomia, and axial skeletal malformations. These observations suggest that *RNF2* nonsense variants may be the genetic basis of a separate RNF2-related developmental disorder. Identification of additional individuals with *RNF2* nonsense variants and functional evaluation will be critical to clarify such a genotype–phenotype relationships and pathogenic mechanisms.

The distinct NDDs caused by *RNF2* and *RING1* (OMIM: 602045) missense variants suggest paralogue-specific functions, despite their seemingly shared molecular mechanisms [35]. In human NPCs, both RNF2 and RING1 are incorporated, without detectable bias as assessed to date, into the different heterogenous PRC1 complexes and exhibit overlapping genome-wide binding profiles [31]. Moreover, hypomorphic RNF2^MS^ and RING1^MS^, encoded by pathogenic missense variants, retain the same genomic binding pattern while impairing H2A monoubiquitination [31]. Yet unlike LUSYAM, RING1-related disorder is characterized by growth restriction, primordial dwarfism and early-onset schizophrenia [35]. These divergent phenotypes, despite molecular parallels, underscore the existence of paralogue-specific developmental roles for H2AK119ub1 placed by RNF2- and RING1- containing PRC1 complexes, despite their molecular parallels. Evolving *RNF2* and *RING1* human genetic findings highlight the need for further investigation into paralogue-specific regulation of H2AK119ub1 dynamics during development.

To elucidate the developmental consequences of *RNF2* missense variants, we generated isogenic hESCs homozygous for the hypomorphic *RNF2* c.292_293AG>CA;p.R98Q allele. The R98Q substitution has been biochemically and structurally shown to impair H2A monoubiquitination, providing a well-defined model to study the impact of disrupted RNF2 catalytic activity [2, 31]. Although the biallelic configuration does not reflect the monoallelic zygosity of LUSYAM *RNF2* missense variants, it enables high-resolution dissection of variant function and highlights aspects of dorsal forebrain differentiation that are sensitive to reduced RNF2-dependent H2AK119ub1. In the *RNF2*^*MS/MS*^ model we detected a striking shift toward ectopic mesenchymal differentiation along the CNC lineage at the expense of the dorsal forebrain identity during early stages of neural differentiation. These findings have translational implications, as the altered dorsal forebrain and neural crest fate trajectories are consistent with many LUSYAM clinical features [29]. The homozygous model establishes a framework for future studies of heterozygous *RNF2* missense variants that affect functions mediated by distinct protein domains, to assess their effects on dorsal forebrain and neural crest development over extended differentiation timelines.

Our multi-omics data support a model in which RNF2-dependent H2AK119ub1 functions as a chromatin barrier that restricts alternative lineage trajectories during neural differentiation. During *RNF2*^*MS/MS*^ dorsal forebrain differentiation we observed a reduction of H2AK119ub1 and H3K27me3 at key mesenchymal transcription factors such as *TWIST1* and *ALX1*, coinciding with their derepression and the activation of mesenchymal gene expression programs. The loss of these repressive marks was associated with ectopic EMT-like transcriptional signatures, consistent with a general increase in transcriptional permissiveness. These findings align with previous mouse ESC studies showing that PRC1 and H2AK119ub1 sustain a deep OFF state of long-lived repression and counteract binding of factors that enable transcription [40]. During *RNF2*^*MS/MS*^ neural differentiation, ectopic expression provides examples where deep repression is undermined by catalytically inactive RNF2^MS^ and reduced H2AK119ub1. More broadly, the reduction of Polycomb histone modifications creates a permissive chromatin environment that allows for transcription factor access associated with differential expression. These findings provide functional evidence linking catalytic dysfunction of RNF2-containing PRC1 with compromised transcriptional plasticity required for lineage fidelity during early human neural development.

Ectopic activation of EMT during *RNF2*^*MS/MS*^ neural differentiation reveals a potential mechanistic convergence between NDDs and cancer. EMT is a well-established driver of cellular plasticity, enabling both neural crest delamination during embryogenesis and promoting metastasis of carcinomas [41]. RNF2 is frequently overexpressed in a broad spectrum of human cancers, including breast, prostate, and bladder carcinomas, where elevated expression is associated with poor prognosis and metastasis [42]. Mechanistically, RNF2 overexpression has been shown to increase global H2AK119ub1 levels, resulting in repression of (E-cadherin), a key epithelial adhesion protein upregulated during EMT [43]. Fewer studies have investigated the pathogenic mechanisms associated with the *RNF2* missense variants detected in oncological samples. TWIST1 induced transcriptional regulation promotes EMT and neural crest migration by upregulating N-cadherin and suppressing E-cadherin, a process similarly hijacked in cancer metastasis [44–46]. The derepression of *TWIST1* and corresponding mesenchymal fate transformation in the *RNF2*^*MS/MS*^ model indicates that both hypo- and hypermorphic RNF2 activity mis-regulates EMT cell biology. This suggests that EMT regulators are a potential node of convergent pathology between NDDs and metastatic *RNF2*-driven malignancies [47]. More broadly, our results highlight the role of RNF2 in maintaining lineage fidelity and demonstrate the utility of developmental models for uncovering chromatin-based mechanisms relevant to cancer biology.

In sum, our findings demonstrate that RNF2-mediated H2AK119ub1 is essential for maintaining neural lineage fidelity during early human neural differentiation. Reduced H2AK119ub1 levels due to catalytically hypomorphic RNF2 missense variants permits ectopic activation of mesenchymal fates. These results not only establish H2AK119ub1 as a critical barrier to inappropriate transcriptional programs but also offer mechanistic insights into the developmental pathogenesis of RNF2-associated NDDs and tumor metastasis.

## Methods

### Clinical Data Acquisition:

Clinical data was collected by clinicians under the IRB: AAA5718 and 16–013231.

### Mapping of mutation and structural modelling to decode the molecular basis of impact of mutation on pathogenesis

The residue conservation of RNF2 among different species was calculated by performing multiple sequence alignment of different species of RNF2 (H. sapiens, M. mulatta, E. caballus, B. taurus, S. scrofa, etc.) and generating a sequence logo diagram through WebLogo, a sequence logo generator [48]. The mutations were mapped on the reported CryoEM structure of human RYBP-PRC1 bound to mononucleosome (PDB: 8PP7) using UCSF ChimeraX v.1.7 [49], an interactive visualization program highlighting the mutations Protein–Protein intramolecular interactions. The protein-protein interaction interfaces were calculated and highlighted on the PRC1 modelled complex using ScanNet [32], a tool for structure-based protein binding sites prediction. The variant effect predictions for RNF2 substitutions were annotated through the variant effect score (VES) is the ESMb1 log likelihood ratio (LRR) of effect scores: (https://huggingface.co/spaces/ntranoslab/esm_variants).

### PRC1 Complex Structure Modelling

The full-length sequence of RNF2 was taken from UniProt id: Q99496. Alphafold3 [50] was used for modelling the structure of PRC1 complex and the sequences corresponding to individual subunits were taken from UniProt. US-align: Universal Structure alignment tool [51] was used for 3D structural alignment of modelled and cryo-EM structure of PRC1 complex and root mean square deviation was calculated.

### Human Embryonic Stem Cell Culture

H9 hESCs (WiCell Lot#WB0090) were cultured feeder free on Matrigel (Corning, Catalog #354234) with mTeSR^™^ Plus (Stem Cell Technologies, Catalog #100–0276). Media was changed every 24 hours. Culture conditions were 37°C and 5% CO_2_. When cells reached ~80% confluency, they were split 1:50. Detachment was achieved through application of Versene (Gibco, Catalog #15040066) for 7 minutes at 37 °C, followed by resuspension and plating in mTeSR^™^ Plus with 10μM ROCK inhibitor (Tocris).

### CRISPR-Cas9 Genome Editing

#### Integrated DNA Technologies (IDT) Alt-R System:

To generate *RNF2*^*MS/MS*^ ESCs (2 independent lines), we employed the IDT Alt-R genome editing protocol. A 35mm plate of H9 hESCs at ~80% confluence was dissociated with Versene, pelleted by centrifugation at 300 rcf for 5 minutes. Oligo sequences to generate crRNA and donor template were designed using the Alt-R gRNA design tool from IDT. Donor oligos were designed to introduce missense changes in addition to asynonymous change to the PAM site. 5μl 100 μM Alt-R CRISPR-Cas9 crRNA was mixed with 5μl 100 μM Alt-R CRISPR-Cas9 trRNA and incubated at 95°C for 5 minutes. The resulting gRNA was mixed with 6.67μl Alt-R S.p. Cas9 Nuclease V3 (IDT, Catalog #1081058) and incubated at room temperature for 20 minutes. The Cas9:gRNA solution was added to 75.6μl electroporation solution (consisting of 62μl human stem cell nucleofector solution and 13.6μl supplement from the Lonza Human Stem Cell Nucleofector Kit 2 VPH-5002), 4μl HDR donor oligo (if generating missense variants), and 4μl 100μM Alt-R Cas9 Electroporation Enhancer. The H9 cell pellet was resuspended in this 100μl CRISPR electroporation cocktail and electroporated in an Amaxa Nucleofector using program A-23. After electroporation, cells were immediately resuspended in mTeSR^™^ Plus containing 1x CloneR^™^ (Stem Cell Technologies, Catalog #05888) and ~0.69mM Alt-R HDR Enhancer V2 (if generating missense variants) and plated on 35mm Matrigel dishes. Media was then changed every 24 hours until colonies were formed. crRNA and donor sequences used (* denotes a phosphorothioate modified oligo): *RNF2* crRNA: (rGrUrCrUrGrGrCrCrUrUrArGrUrGrArUrCrUrUrUrGrUrUrUrUrArGrArGrCrUrArUrGrCrU, targeted DNA sequence GTCTGGCCTTAGTGATCTTT) *RNF2* c.292_293AG > CA;p.R98Q Donor: (CAAAGAATGTCCTACCTGTCGGAAAAAACTAGTTTCAAAACAATCACTAAGGCCAG ACCCAAACTTTGATGCACTCATCAGCA)

### Sanger Sequencing of CRISPR-Cas9 Clones

Colonies were dissociated and plated at low densities by serial dilution onto 60mm Matrigel plates to form clonal colonies. Single colonies were picked and cultured in a 48-well plate. Once confluent, 48-well plates were split and DNA was extracted for PCR amplification and Sanger sequencing (Eurofins Genomics) to confirm genotypes. Two separate clones were identified for each genotype and expanded for use. PCR and sanger primers: *RNF2* fwd: 5’ GGTATAGAGGGTTTGAGGTTTCC 3’ *RNF2* rev: 5’ TCCTGGCTAATACTCTCTCTTG 3’

### Neural Differentiation of hESCs to Neural Rosettes and NPC Monolayer Cultures

To generate rosettes of NPCs from ESCs, we first formed aggregates of ESCs by sedimentation as previously described [52]. Briefly, 600 cells in 30ul droplets containing mTeSR^™^ Plus with 10μM ROCK inhibitor were added to each well of a 96-well non-adherent V-bottom plate (SBio, Catalog #MS-9096VZ). 24 hours later, cultured hESCs were then differentiated to NPCs and dorsal forebrain organoids via a previously described dual SMAD inhibition protocol [53]. First, neural induction is initiated by changing ESC culture media to N2 + SMADi medium [49.5mL DMEM/F12 (Invitrogen), 500ul N2 supplement (Invitrogen), 83ul 600uM Dorsomorphin (Tocris), and 5ul 20mM A 83–01 (Tocris)]. Medium was changed every other day until day 7, when it was switched to N2+B27+Dorsomorphin+FGF medium [49mL DMEM/F12, 250 ul N2 supplement, 500ul B27 supplement (Invitrogen), 83ul 600uM Dorsomorphin, and 100μl of 10ng/ul bFGF] and aggregates are transferred onto 35mm Matrigel plates using a 1000μl pipette. Medium was then changed every other day until day 14, when medium was switched to a maintenance N2+B27+ bFGF media [49mL DMEM/F12, 250 ul N2 supplement, 500ul B27 supplement (Invitrogen), and 100μl of 10ng/ul bFGF]. To generate NPCs from visible clusters of NPC rosettes, rosettes were removed from the dish manually with a pipette tip under the guidance of an Invitrogen^™^ EVOS^™^ microscope and subjected to chemical dissociation via 10-minute Accutase incubation (Invitrogen). Dissociated cells were pelleted at 300 rpm for 5 minutes and plated in N2+B27+ bFGF media on 15ug/mL poly-L-ornithine (Sigma) and 10ug/mL laminin (Sigma) coated dishes.

### Nuclear Extractions

Nuclei from 2.5–5 ×10^6 cells were placed in trypsin for 2 min at 37°C then neutralized with DMEM. Cells were spun down for 30 sec at 600 rpm and resuspended in 1ml PBS (Gibco, 14190–144). Cells were then spun for 5 min at 200 × g at 4°C. After removal of PBS, cells were resuspended in 500μl cold EZ nuclei lysis buffer (Sigma, NUC101–1KT) with 1μl protease inhibitor, and dounce homogenized in 200μl EZ buffer. Cells were then spun at 500 × g in 4 °C for 5 min. Remove supernatant and resuspended in 400μl EZ nuclei buffer (Sigma, NUC101–1KT). Centrifuged again at 500 × g 4°C for 5 min. Nuclei were resuspended in 40ul wash buffer. Once counted, nuclei were then used for CUT&RUN.

### CUT&RUN

CUT&RUN experiments were conducted utilizing the EpiCypher CUTANA^™^ ChIC/CUT&RUN kit v3 protocol (Epicypher, Durham NC). Nuclei were captured with BioMagPlus Concanavalin A beads and incubated with 1 μg primary antibody in 200 μL wash buffer for 2 h. Primary antibodies used were: RING1B (Cell Signaling, D22F2), Ring1A (Cell Signaling, 2820S), H2AK119ub1 (Cell Signaling, D27C4), H3K27me3 (Abcam, AB6002) and IGG (Epicypher). After 24 hours, unbound antibody was washed away with 100μL wash buffer twice. Then pA-MN was added at 2.5μL/reaction and incubated for 10 min at room temperature. The nuclei were then washed and resuspended in 200μL cell permeable buffer for two washes. Cells were resuspended with 50ul cold cell perm buffer. 1ul of 100mM CaCl_2_ was next added to activate the enzyme. Next, 0.5 ng of E. coli spike-in DNA were added to each sample. The reaction was carried out at 0 °C and stopped by 150μL of 2X STOP buffer (200 mM NaCl, 20 mM EDTA, 50μg/mL RNase A, and 40μg/mL glycogen). Protein-DNA complex was released by centrifugation and digested by proteinase K at 50 °C overnight, followed by DNA precipitation by ethanol. The pellet was washed with 70% ethanol and dissolved in 25 μL 0.1× TE (1 mM Tris-HCl pH 8.0, 0.1 mM EDTA).

### CUT&RUN Library Prep

Library preparation was done with the NEBNext Ultra II DNA Library Prep Kit. Briefly, 6 ng of CUT&RUN DNA were treated with endprep module at 20 °C for 30 min and 50 °C for 1 h. Ligation was performed by adding 5 pmol of NEB adapter and ligation mix and incubated at 20 °C for 15 min. To clean up the reaction, add 1.75× volume of Agencourt AMPure XP beads (Beckman Coulter) to capture short ligation products. PCR amplification was performed for 12 cycles. The resulting libraries were purified with 1.2× volume of AMPure beads then analyzed and quantified by Qubit and Tapestation. For H2AK119ub1, paired-end sequencing was performed by the University of Michigan Advanced Genomics Core using a Next Seq P3 100 cycle kit (Illumina) and sequenced on a NextSeq platform with an average of 23 million reads/sample. For RING1 and RNF2, paired-end sequencing was performed by the University of Michigan Advanced Genomics Core using a Next Seq P2 100 cycle kit (Illumina) and sequenced on a NextSeq platform with an average of 12 million reads/sample.

### Data Processing and Normalization

FASTQ files were aligned to the human reference genome hg38 using bwa-aln and paired reads were merged into SAM file format using bwa-sampe. Aligned SAM files were converted to BAM format with samtools. BAM files were then cleaned, sorted by coordinate, and PCR duplicates were removed using picard-tools/2.8.1 (https://broadinstitute.github.io/picard/). Unmapped reads were then filtered out using samtools. Reads aligning to annotated blacklist regions (ENCFF356LFX) were removed using the bedtools intersect function. Reads with map quality scores below 30 were removed using samtools. Correlation between of CUT&RUN signal at PRC1 target loci across biological replicates was determined using deepTools functions multiBamSummary and plotCorrelation [54]. All biological replicates were highly correlated, with all having a Pearson’s correlation coefficient > 0.9, and were merged for subsequent analysis. Data were entered into GraphPad Prism to generate heatmap plots of correlations. Merged BAM files were then converted to BIGWIG format for visualization using the coverage function in the R package rtracklayer [55]. When converting to BED format, merged BAM files were normalized to Counts Per 10 Million mapped reads (CPM). CUT&RUN tracks were then visualized using the integrated genome viewer [56].

### Identification of PRC1 Target Domains

The 4255 PRC1 targets highlighted in our analysis were determined through identification of *RING1* and *RNF2* CUT&RUN peaks that were present in both biological replicates of wild type NPCs. First, the MACS2 callpeak function used to call peaks on wild type RING1 and RNF2 samples relative to their IGG controls with the following parameters: -t sample.bam, -c IGG_control.bam, -f BAMPE, -g 2913022398, -q 5.00e-02, --broad [57]. Sample.bam files were the merged bam file from two highly correlated biological replicates. IGG_control.bam files were the merged IGG files from the two experiments corresponding to the Sample.bam files. Resulting peaks were then analyzed using the R packages GenomicRanges and rtracklayer to identify 305 RING1 and 4239 RNF2 peaks present in both biological replicates [55, 58]. Of the 305 RING1 peaks, 289 were also present in the RNF2 peaks. BED files were exported from R, and bedops -m was used to combine BED files containing the RING1 and RNF2 peaks reproduced in both biological replicates. Bedtools merge function was then used to consolidate overlapping peaks. This resulted in the identification of 4255 RING1 and/or RNF2 bound loci that we define as PRC1 targets.

### Generation of CUT&RUN heatmaps and profile plots

For analysis at 4255 PRC1 target loci, deeptools computematrix was used with parameters scale-regions, -p 10, -b 5000, -a 5000 [54]. Profile plots were generated using deeptools plotProfile. Heatmaps were generated using deeptools plotHeatmap, and loci were clustered into three groups using the -kmeans option. Input bigWig files were normalized to counts per 10 million mapped reads (CPM).

### Bulk RNA sequencing and Differential Expression Analysis

NPC monolayer cultures from three biological replicates were generated for each genotype across two independent differentiations. Cultured NPCs at approximately 80% confluence in 24-well plates were harvested for RNA using a PureLink^™^ RNA Mini Kit (Invitrogen, #12183018A). Lysates were homogenized by passage 10 times through a 21-gauge needle. RNA library preparation was performed by performed by Azenta Life Sciences with PolyA selection (Illumina). Libraries were sequenced on an Illumina 2×150bp instrument, targeting approximately 15 million paired-end reads per sample. FASTQ files were aligned to HG38 refGene transcripts using STAR [59]. Resulting gene counts were analyzed for statistically significant changes using DESeq2, with the batch effect (independent hESC differentiation to NPCs) as a covariate [60]. MA plots were generated from DESeq2 results in R.

### CUT&RUN Correlation Analysis:

To correlate CUT&RUN signal across genes, a bed file containing annotated RefSeq transcripts aligned by UCSC was first downloaded and the genome start and stop locations of transcripts was used as regions of interest (hg38.refGene.gtf, downloaded (https://hgdownload.soe.ucsc.edu/goldenPath/hg38/bigZips/genes/). To avoid counting a gene multiple times for each of its annotated isoforms in the RefSeq database, the AGAT agat_sp_keep_longest_isoform.pl function was used to keep only the longest isoform in the GTF file [61]. Next, this GTF file was loaded into R using the read.delim base R function, and rows without the indicator “transcript” in the gene column were removed in order to eliminate redundant overlapping regions such as internal annotated introns and exons. A bed file containing the chromosome number, start location, and end location for each transcript was then using the base R function write.delim and used for analysis of transcription start sites. This file was then read into R using the CHIPseeker readPeakFile function. Counts from BAM files containing H2AK119ub1 and H3K27me3 reads were then aggregated across gene regions using the summarizeOverlaps function in the R GenomicAlignments package [58]. Transcripts less than 500bp in length were removed to reduce noise in the downstream analysis. Count numbers for each gene were then divided by the length of the gene in base pairs to normalize each gene’s count frequency to length. The resulting length-normalized CUT&RUN counts per gene were plotted in scatterplots using ggplot2.

To visualize the correlation between H2AK119ub1 and H3K27me3, the length-normalized signal per gene was correlated for wild type and *RNF2*^*MS/MS*^. BED files containing the 4255 PRC1 target loci annotated by k-means cluster (generated using deeptools plotHeatmap function as described above) were loaded into R and annotated with gene symbols using CHIPseeker to generate a list of direct PRC1 target genes and their associated cluster (TxDb.Hsapiens.UCSC.hg38.knownGene database and annotations from org.Hs.eg.db) [62–64]. These lists of genes within each k-means cluster were cross-referenced to the data to highlight genes on the correlation plot with the color of their corresponding cluster using ggplot2.

To identify loci of disproportionately decreased H2AK119ub1 or H3K27me3, correlations were plotted for H2AK119ub1 or H3K27me3 signal between wild type and *RNF2*^*MS/MS*^. A linear regression was performed using the R lm() function and residual values for each gene were calculated using the R rstandard() function. The 5% most decreased of genes for H2AK119ub1 or H3K27me3 in *RNF2*^*MS/MS*^ cells were identified using the R quantile() function. The list of genes that represented the 5% most decreased for H2AK119ub1 and H3K27me3 in *RNF2*^*MS/MS*^ cells were then tested for overlap with the differentially expressed genes from bulk RNA sequencing. To determine if these lists shared more genes than would be expected by chance, bootstrapping was performed with 100,000 iterations selecting the same number of genes randomly and assessing the percent overlap each time. Results were considered statistically significant if the observed percent of overlap represented a value greater than the top 5% of simulations.

To understand the relationship between wild type and RNF2 occupancy at TWIST1 and SOX2 binding sites for both H2AK119ub1 and H3K27me3, the normalized signal per gene was correlated between the variables. The list of TWIST1 and NOTCH1 targets, we utilized the transcription factor database TFLink (https://tflink.net/) [65]. Text files with the lists were then loaded into R. The 5% most decreased of genes for H2AK119ub1 or H3K27me3 in *RNF2*^*MS/MS*^ cells were then tested for overlap with differentially expressed genes from bulk sequencing. We performed bootstrapping with 100,000 iterations. In addition, we calculated the absolute and relative fold changes between the genotypes and graphed them as boxplots.

### Single Cell RNA-sequencing and data integration

Three replicates, each containing approximately 12 neural rosettes in a 60mm Matrigel plate, were differentiated as described above. Cells were dissociated with Accutase (Invitrogen) for 10 minutes at 37°C and passed through a 70μm cell strainer. Cells were then fixed using the Fix & Perm Buffer (10x Genomics PN-2000517) with 4% formaldehyde for 1 hour at room temperature. Finally, cells were resuspended in the Quench Buffer (10x Genomics PN-2000516) and submitted to the University of Michigan Advanced Genomics Core for library preparation using the 10x scRNA Flex kit. Libraries were sequenced on a Illumina NovaX 10B 300 cycle instrument. This pool was subjected to 151bp paired-end sequencing according to the manufacturer’s protocol (Illumina NovaSeqXPlus). BCL Convert Conversion Software v4.0 (Illumina) was used to generate de-multiplexed Fastq files. Fastq files were converted to single cell gene expression matrices using the 10x Genomics Cellranger pipeline to generate a count output [66]. We excluded the poor-quality cells in the gene-cell data matrix using the Seurat package (v5.0.1) for the samples. Cells with a number of features less than 2 standard deviations below or more than two standard deviations above the average number of features for each sample were removed. Cells with greater than 10% mitochondrial genes were also removed. We next normalized the data using the Seurat (v5.0.1) sctransform function, which employs a regularized negative binomial regression with cell sequencing depth as a covariate [36]. The 3000 most variable features across all samples were identified using the Seurat function “SelectIntegrationFeatures” [67]. We next used these variable genes to integrate the samples using the FindIntegrationAnchors and IntegrateData functions of Seurat (v5.0.1). For downstream differential expression analysis, we normalized the raw unique molecular identifier (UMI) counts in each sample for each cell by dividing each feature count by the total counts for that cell multiplied by 10,000. This was then natural-log transformed using log1p to generate depth normalized counts for all features and used to compare gene expression across groups of cells.

For dimensionality reduction, visualization and clustering, we used the Seurat package (v5.0.1) to perform dimensionality reduction. We used the integrated and normalized data as the input to the RunPCA function of Seurat (v5.0.1) to compute the first 30 PCs. Visualizations in a two-dimensional space were done using RunUMAP function of Seurat (v5.0.1) for the integrated data using 20 PCs. We performed a graph-based clustering approach using FindNeighbors (using dimensions 1:20) and FindClusters (using resolution 0.5) functions of Seurat (v5.0.1). We then collected cluster marker genes using the Wilcoxon rank-sum test between the cells in a single cluster and all other cells using the Seurat (v5.0.1) FindAllMarkers function with log fold change threshold of at least 0.25. To assign identities to clusters, we cross-referenced the marker genes with previously described cortical and mesenchymal cell marker genes [68–73]. The expression of cell type markers was visualized using the FeaturePlot function in Seurat. The number of cells in each annotated cell type were noted for each sample and used to calculate the proportion of cells in each sample from each cell type.

### Pseudotime analysis

We used Monocle 3 to conduct single-cell pseudotime analysis [74]. First, data was imported from the Seurat object using the as.cell_data_set function. Next, size factors were estimated using the estimate_size_factors function. Cells were clustered within Monacle 3 with the cluster_cells function, using the UMAP reduction method. The learn_graph function was then applied, with use_partition = TRUE. Next, the order_cells function was used, with the UMAP reduction method and selecting cells in NPC clusters as the root nodes. Finally, plot_cells was used to visualize the trajectory. To isolate the neurodevelopmental and mesenchymal trajectories, a subset of the Monocle object was manually selected using the Monocle3 choose_graph_segments function. To visualize changes in gene expression across pseudotime, the Monocle3 plot_genes_in_pseudotime function was used and the pseudotime and expectation values were extracted and plotted using the ggplot2 geom_line function.

### RNA Velocity Methods Section

FASTQ files were generated using Cell Ranger pipeline provided by the University of Michigan Advanced Genomics core. Genome annotations from GRCh38 were used to count for splices and unspliced mRNA in each single cell. Loom files were then generated from the velocyto package using default parameters with human genome assembly GRCh38 as the annotation [37]. Python package scVelo (v.0.2.2, https://scvelo.readthedocs.io) was then used to perform RNA velocity analysis using dynamical modelling [37]. The function ‘scv.pl.velocity_embedding_stream’ was used to project RNA velocities onto UMAP plots. All default parameters were used unless noted.

### Western Blot Analysis

NPCs were washed in PBS and subsequently homogenized in RIPA buffer supplemented with protease inhibitor cocktail and phosphatase inhibitor cocktail 3 obtained from Sigma-Aldrich (P8340 and P0044; St Louis, MO, USA). Protein concentrations were determined using Pierce^™^ BCA Protein Assay Kits (Thermo, 23225). Cell lysates were separated by electrophoresis on 4–20% Tris-Glycine gels and transferred to PVDF membrane using iBlot 2 PVDF Regular Stacks (Invitrogen IB24001) transferred on an Invitrogen iBlot 2 Gel Transfer Device (IB21001). The PVDF membrane was blocked with 5% milk in TBST and incubated with primary antibodies diluted in 5% milk in TBST. Primary antibodies used were: anti-ubiquityl-Histone H2A (Cell Signaling #8240, 1:2000), anti-Vinculin (Cell Signaling, E1E9V, 1:1000), and anti-RNF2 (Cell Signaling # #5694, 1:1000). Donkey anti-rabbit HRP-conjugated (Cytiva, NA9340V, 1 to 5000) and goat anti-mouse HRP-conjugated (Invitrogen, 32430, 1 to 10000) were used for 1h incubation in 5% milk TBST at room temperature. Antibody incubation and chemiluminescence detection were performed using SuperSignal^™^ West Femto Maximum Sensitivity Substrate according to manufacturer’s instruction [ThermoFisher Scientific, cat no. 34095].

### Immunohistochemistry

Neural rosettes that were differentiated as described above were fixed in 4% paraformaldehyde (PFA) at either at day 14 or 21 of differentiation. Cells were kept in 4% PFA at 4°C overnight. Cells were then preserved by submersion in 15% then 30% sucrose solutions. Next, samples were cryopreserved by embedding in OCT cryosectioning media (Tissue-Tek, Torrance, CA). Embedded cells were cryosectioned at 13 μm. Sections were incubated with PBS for 15 min to wash away OCT. For antibodies that required antigen retrieval, cryosections were heated in 10 mM Sodium citrate for 20 minutes at 95°C followed by incubation at room temperature for 20 minutes. Incubation with a normal donkey serum blocking buffer [5% NDS (Jackson ImmunoResearch), 0.1% Triton X-100, 5% BSA] was performed for 1 hour. Sections were stained with primary antibodies in blocking buffer at 4°C overnight, washed with PBS, and stained with secondary antibodies at room temperature for 1 hour. Slides were washed with PBS, incubated with DAPI for 5 minutes and cover slipped with ProLong Gold. Images were acquired with a NIKON N-SIM + A1R microscope and processed with LAS X software. The following antibodies and dilutions were used: rabbit SOX2 (invitrogen PA1–16968), goat DCX (C-18) (Santa Cruz: sc-8066), and mouse TWIST1 [Twist2C1a] (ab50887). AlexaFluor-conjugated secondaries were Alexa Fluor^™^ 488 Donkey anti-goat IgG Secondary Antibody (ThermoFisher Catalog # A-11055), Alexa Fluor^™^ 555 Donkey anti-Mouse IgG Secondary Antibody (ThermoFisher Catalog # A-31570), and Alexa Fluor^™^ 647 Donkey anti-rabbit IgG Secondary Antibody (ThermoFisher Catalog # A-31573).

## Supplementary Files

This is a list of supplementary files associated with this preprint. Click to download.

- RNF2VariantDataSupplementalData.xlsx
- RNF2SupplementalData.pdf

## Figures and Tables

**Table 1:** Summary of main clinical features in individuals with monoallelic *RNF2* variants.

		Individual #1*	Individual #2*	Individual #3	Individual #4	Individual #5	Individual #6
Age at last evaluation		11 years	3.5 years	6 years	21 months	4 years	7 years
Sex		F	F	M	M	M	F
RNF2 variant (NM_007212.3)		c.209G>A (p.R70H)	c.246T>G (p.S82R)	c.245G>T;(p.S82I)	c.245G>T (p.S82I)	c.796A>T (p.R266T)	c.472C>T (p.R158*)
Inheritance		*De novo*	*De novo*	*De novo*	*De novo*	*De novo*	Unknown
Prenatal findings		IUGR, oligohydramnios	IUGR	Abnormal cisterna magna and septum pellucidum, unilateral hydronephrosis, polyhydramnios	IUGR, single umbilical artery, elevated fetal dopplers	Abnormal cardiac scan @ 20w	Single umbilical artery
Birth parameters	Length	48.3 cm (88%ile)	44.5 cm (4%ile)	53 cm (99%ile)	47 cm (54%ile)	Not available	Not available
	Weight	2381 g (24%ile)	2330 g (4%ile)	3800 g (96%ile)	2220 g (9%ile)	2190 (48%ile)	2580 (6%ile)
	OFC	29 cm (2%ile)	31.5 cm (4%ile)	38.5 cm (99%ile)	33 cm (29%ile)	Not available	Not available
	GA	36 weeks	39 weeks	38–1/7 weeks	37+4/7 weeks	Not available	Not available
Dysmorphic features		+	+	+	+	+	+
Current growth parameters	Height	141.8 cm (25%ile)	93 cm (14%ile)	104.5 cm (<1%ile)	86.4 cm (69%ile)	101 cm (39%ile)	107.8 cm (<1%ile)
	Weight	36.3 kg (25–50%ile)	13.4 kg (20%ile)	16.32 kg (<1%ile)	12.8 kg (66%ile)	14.8 kg (21%ile)	18 kg (4%ile)
	OFC	50.4 cm (1%ile)	48 cm (10–25%ile)	53.5 cm (92%ile)	48.3 cm (51%ile)	1%ile	50 cm (15%ile)
Developmental delay/Intellectual disability		Severe GDD & ID	Severe GDD & ID	GDD & ID	GDD & ID	Mod-severe GDD & ID	Learning disability
Abnormal tone		Hypotonia	Hypotonia	Hypotonia, spasticity	Hypotonia, spasticity, global weakness	-	Hypotonia
Other neurological features		Seizures, hyperreflexia	Seizures, hand stereotypies	-	Hyperkinetic movements, possible dystonia	Focal epilepsy, reduced reflexes in lower limbs	Complex tethered cord, lipomyelomeningocele
Brain imaging abnormalities		Diffuse loss of WM	Possible delayed myelination @ 3m	-	Delayed myelination	-	Chiari I, hypoplastic left cochlea, absent cochlear nerve
Ophthalmologic		Strabismus, astigmatism	Strabismus	Myopia, astigmatism	Esotropia, anisocoria, CVI	Strabismus, left hypermetropia, 4^th^ CN palsy	Amblyopia, horizontal gaze palsy
GI/Feeding difficulties		FTT, omphalomesenteric duct cyst	Dysphagia, dysmotility, GERD	FTT, EoE, constipation	Dysphagia, GERD	FTT, GERD	FTT
Hearing/ENT		Recurrent otitis media	-	Eustachian tube dysfunction, chronic serous otitis media	Recurrent otitis media	Mod-severe bilateral CHL	Left severeprofound HL, Hx recurrent otitis media
Musculoskeletal		Not noted	Not noted	Coxa valga, metatarsus adductus, neuromuscular scoliosis, plagiocephaly	Plagiocephaly	Hypermobility	Severe congenital scoliosis, torticollis, plagiocephaly, cystic mandible lesion, 5^th^ finger clinodactyly
Cardiovascular		Mild tricuspid regurgitation	-	-	Atrial septal defect	Truncus arteriosus, dilated aorta	Small secundum atrial septal defect
Other		Pilomatrixomas on cheek and ear	Umbilical hernia	Reactive airway disease, delayed bone age, yellow discoloration of skin around nose	Resolved left hydronephrosis, sleep apnea, chronic respiratory failure during sleep	Severe TMJ tightness/diffic ult airway, intermittent facial swelling, small patch of alopecia	Horseshoe kidney, grade II vesicoureteral reflux, high pain tolerance, hemifacial microsomia

* Data obtained from Luo et al., 2021

IUGR = intrauterine growth restriction, SGA = small for gestational age, OFC = occipital frontal circumference, GDD = global developmental delay, FTT = failure to thrive, TMJ = temporomandibular joint, WM = white matter, CVI = cortical visual impairment, CN = cranial nerve, EoE = eosinophilic esophagitis, CHL = conductive hearing loss, HL = hearing loss, Hx = history. Percentiles based on World Health Organization growth charts.
